# Karyotype Characterization of Nine Periwinkle Species (Gastropoda, Littorinidae)

**DOI:** 10.3390/genes9110517

**Published:** 2018-10-23

**Authors:** Daniel García-Souto, Sandra Alonso-Rubido, Diana Costa, José M. Eirín-López, Emilio Rolán-Álvarez, Rui Faria, Juan Galindo, Juan J. Pasantes

**Affiliations:** 1 Departamento Bioquímica, Xenética e Inmunoloxía, Universidade de Vigo, E-36310 Vigo, Spain; danielgarciasouto@gmail.com (D.G.-S.); salonsorubido@gmail.com (S.A.-R.); rolan@uvigo.es (E.R.-A.); galindo@uvigo.es (J.G.); 2 CIMUS Biomedical Research Institute, University of Santiago de Compostela, E-15706 Santiago de Compostela, Spain; 3 Systems Biotechnology Group, Department of Applied Biocatalysis, CSIC-Institute of Catalysis and Petrochemistry, C/Marie Curie 2, E-28049 Madrid, Spain; 4 CIBIO, Centro de Investigação em Biodiversidade e Recursos Genéticos, InBIO, Laboratório Associado, Campus Agrário de Vairão, Universidade do Porto, 4485-661 Vairão, Portugal; diana.costa.c0805147@gmail.com (D.C.); r.macieiradefaria@sheffield.ac.uk (R.F.); 5 Department of Biological Sciences, Florida International University, Miami, FL 33199, USA; jeirinlo@fiu.edu; 6 CIM-UVIGO, Centro de Investigación Mariña, Universidade de Vigo, E-36331 Vigo, Spain; 7 Department of Animal and Plant Sciences, University of Sheffield, Sheffield S10 2TN, UK

**Keywords:** periwinkles, chromosome, fluorescent in situ hybridization, histone genes, ribosomal RNA genes

## Abstract

Periwinkles of the family Littorinidae (Children, 1834) are common members of seashore littoral communities worldwide. Although the family is composed of more than 200 species belonging to 18 genera, chromosome numbers have been described in only eleven of them. A molecular cytogenetic analysis of nine periwinkle species, the rough periwinkles *Littorina arcana*, *L. saxatilis*, and *L. compressa*, the flat periwinkles *L. obtusata* and *L. fabalis*, the common periwinkle *L. littorea*, the mangrove periwinkle *Littoraria angulifera*, the beaded periwinkle *Cenchritis muricatus*, and the small periwinkle *Melarhaphe neritoides* was performed. All species showed diploid chromosome numbers of 2n = 34, and karyotypes were mostly composed of metacentric and submetacentric chromosome pairs. None of the periwinkle species showed chromosomal differences between male and female specimens. The chromosomal mapping of major and minor rDNA and *H3* histone gene clusters by fluorescent in situ hybridization demonstrated that the patterns of distribution of these DNA sequences were conserved among closely related species and differed among less related ones. All signals occupied separated loci on different chromosome pairs without any evidence of co-localization in any of the species.

## 1. Introduction

The family Littorinidae (Children, 1834) comprises more than 200 species of periwinkles grouped in 18 genera that are common members of marine intertidal communities around the world. Although the systematics and evolution of the Littorininae have been widely studied [1,2,3,4], there is still some degree of taxonomic confusion due in part to the high phenotypic polymorphism showed by some of the taxa. This, together with the existence of species complexes where gene flow and/or incomplete lineage sorting has been detected between closely related species, makes their identification difficult for downstream evolutionary and ecological studies. The best known of these complexes of species are those composed by the rough periwinkles *Littorina arcana* (Hannaford-Ellis, 1978), *L. compressa* (Jeffreys, 1865), and *L. saxatilis* (Olivi, 1792), and those including the flat periwinkles *L. fabalis* (W. Turton, 1825) and *L. obtusata* (Linnaeus, 1758).

Cytogenetic analyses, mostly limited to the description of chromosome numbers, have been published for a total of 11 species of the family Littorinidae (Table 1) [5,6,7,8,9,10,11,12,13,14,15]. Five of these species belong to the genus *Echinolittorina* [*E. hawaiiensis* (Rosewater & Kadolsky, 1981), *E. miliaris* (Quoy & Gaimard, 1833), *E. punctata* (Gmelin, 1791), *E. radiata* (Souleyet, 1852), and *E. subnodosa* (Molina, 1782)], four to the genus *Littorina* [*L. brevicula* (Philippi, 1844), *L. keenae* (Rosewater, 1978), *L. obtusata*, and *L. saxatilis*], and the remaining two are *Littoraria strigata* (Philippi, 1846) and *Melarhaphe neritoides* (Linnaeus, 1758). Karyotype compositions have been described for *L. saxatilis* [7,10,11], *M. neritoides* [6,9,11], *E. subnodosa* [13], and *L. keenae* [15], and the existence of sex chromosome determination systems has been proposed for both *M. neritoides* (X0) [9,11] and *L saxatilis* (XY) [12]. Furthermore, the presence of closely associated repetitive DNA sequences on a single chromosome location has been described in *M. neritoides* [14].

In this work, we characterized the chromosomes of nine periwinkle species by 4′,6-diamidino-2-phenylindole (DAPI) staining and fluorescent in situ hybridization (FISH) mapping of major (45S) rDNA, minor (5S) rDNAs, and *H3* histone gene clusters. A fragment of the mitochondrial cytochrome c oxidase subunit I (COI) gene was further amplified and sequenced to add independent genetic information for species identification.

## 2. Materials and Methods

Periwinkles were collected at the localities indicated in Table 2. Attending to shell and internal morphology criteria, the specimens were preliminarily identified as *Littorina arcana* (Hannaford-Ellis, 1978), *Littorina saxatilis* (Olivi, 1792), *Littorina compressa* (Jeffreys, 1865), *Littorina fabalis* (W. Turton, 1825), *Littorina obtusata* (Linnaeus, 1758), *Littorina littorea* (Linnaeus, 1758), *Littoraria angulifera* (Lamarck, 1822), *Cenchritis muricatus* (Linnaeus, 1758), and *Melarhaphe neritoides* (Linnaeus, 1758). The nomenclature used for the taxa follows the World Register of Marine Species database [16]. Representatives of the two sympatric ecotypes of *L. saxatilis* [4] and the three morphotypes/ecotypes described for the Iberian Peninsula *L. fabalis* [17] were included.

Periwinkles were transported to the laboratory and processed following a combination of methods previously described for mollusks [18,19,20]. After an overnight colchicine (0.005%) treatment, periwinkles were euthanized and their muscular feet were dissected and preserved in absolute ethanol. The rest of the soft tissues were immersed in 50% (3 h) and 25% (3 h) sea water containing colchicine (0.05%) prior to fixation with ethanol/acetic acid. Small pieces of fixed gills and gonads were immersed in 60% acetic acid and disaggregated to obtain cell suspensions that were spread onto preheated slides [21,22].

Genomic DNA, extracted with the EZNA Mollusc DNA Kit (Omega Bio-Tek, Norcross, GA, USA), was used to amplify the *H3* histone gene, 5S rDNA, and 28S rDNA in a GeneAmp PCR system 9700 (Applied Biosystems, Foster City, CA, USA). PCRs were performed in 20 μL total volume containing 1× PCR buffer, 2.5 mmol/L MgCl_2_, 50 ng DNA, 0.5 mmol/L each dNTP, 1 μmol/L each primer, and 1 U BIOTAQ DNA polymerase (Bioline, London, UK). *H3* histone gene and 5S rDNA probes were labeled by supplementing the PCR mixtures either with 20 μM biotin-16-dUTP (Roche Applied Science, Penzberg, Germany) or 5 μM digoxigenin-11-dUTP (10× DIG Labeling Mix, Roche Applied Science), whilst 28S rDNA probes were labeled employing a nick translation kit (Roche Applied Science) [23,24,25,26].

Chromosome preparations were stained with DAPI (0.14 μg/mL) for 8 min, air dried, mounted with antifade (Vectashield, Vector Laboratories Inc., Burlingame, CA, USA), and photographed using a Nikon Eclipse-800 microscope equipped with a DS-Qi1Mc CCD camera (Nikon, Tokyo, Japan) controlled by the NIS-Elements software (Nikon). After visualization and photography, single and/or double FISH experiments using *H3* histone gene, 5S rDNA, and/or 28S rDNA probes were performed following previously published methods [23,24,25,26]. In short, chromosome preparations were digested with RNase and pepsin, fixed with formaldehyde and denaturated in 70% formamide at 69 °C (2 min). After overnight hybridization in 50% formamide at 37 °C, chromosome preparations were washed at 45 °C with 50% formamide and 1× SSC. Biotin-labeled probes were detected with fluorescein avidin and biotinylated anti-avidin (Vector Laboratories) whereas digoxigenin-labeled probes were detected with mouse antidigoxigenin, goat antimouse rhodamine, and rabbit antigoat rhodamine (Sigma–Aldrich, St. Louis, MO, USA). Chromosome preparations were counterstained with DAPI, mounted with antifade and the previously recorded metaphase plates photographed again. Chromosome preparations subjected to double FISH experiments were then rehybridized using a third probe, and the same metaphase plates were photographed once more.

Karyotype analyses were performed in a minimum of 10 specimens (5 females, 5 males) from each periwinkle species. A minimum of 10 metaphase plates per specimen was analyzed. For each periwinkle species, the best 10 metaphase plates showing FISH signals, obtained from different individuals, were employed to build karyotypes. Measurements of short and long arm lengths were taken, and relative lengths and centromeric indices from each chromosome pair were determined for each metaphase plate. Mean values per chromosome pair and species were also calculated.

The morphological identification of the specimens was confirmed by amplifying and sequencing a fragment of the mitochondrial cytochrome c oxidase subunit I (*COI*) gene [27,28]. All sequences were deposited in the NCBI GenBank database under the accession numbers MH809396 to MH809424 (Supplementary materials Figure S1).

## 3. Results

All male and female specimens of the nine periwinkle species showed diploid chromosome numbers of 2n = 34 and karyotypes characterized by a superabundance of meta/submetacentric chromosome pairs (15 to 17).

**Figure 1:**
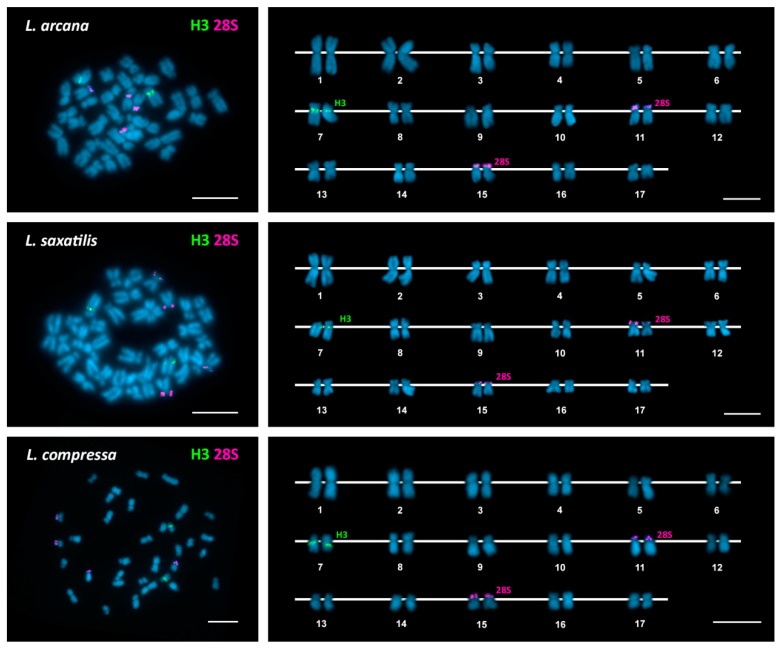
Chromosomal mapping of major rDNA and histone gene clusters in rough periwinkles. Fluorescent in situ hybridization mapping of *H3* histone gene (H3, green) and major rDNA (28S, magenta) probes to mitotic chromosomes of the rough periwinkles *Littorina arcana*, *Littorina saxatilis*, and *Littorina compressa* counterstained with 4′-6-diamidino-2-phenylindole (DAPI). Scale bars, 5 μm.

**Figure 2:**
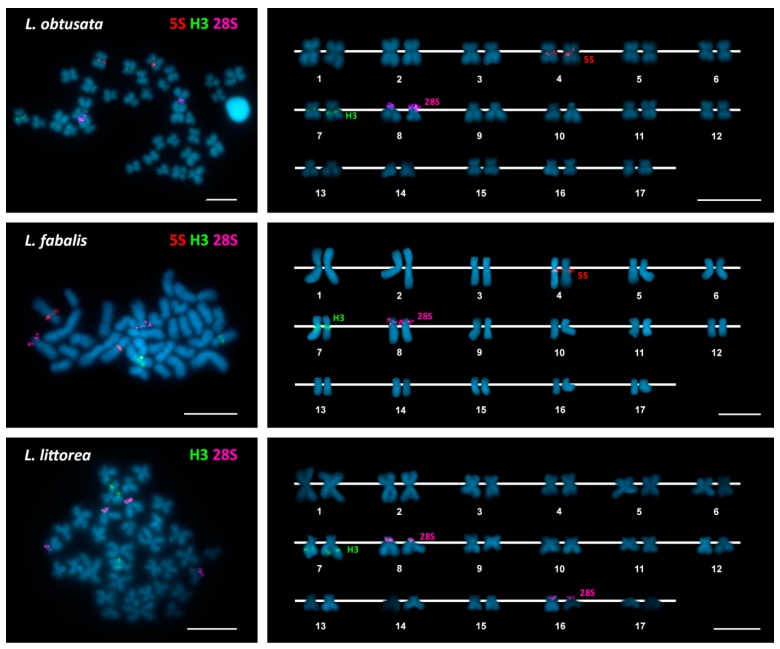
Chromosomal mapping of rDNA and histone gene clusters in flat and common periwinkles. Fluorescent in situ hybridization mapping of minor rDNA (5S, red), *H3* histone gene (H3, green) and major rDNA (28S, magenta) probes to mitotic chromosomes of the flat periwinkles *Littorina obtusata* and *Littorina fabalis* and the common periwinkle *Littorina littorea* counterstained with 4′-6-diamidino-2-phenylindole (DAPI). No minor (5S) rDNA signals were detected in the common periwinkle. Scale bars, 5 μm.

**Figure 3:**
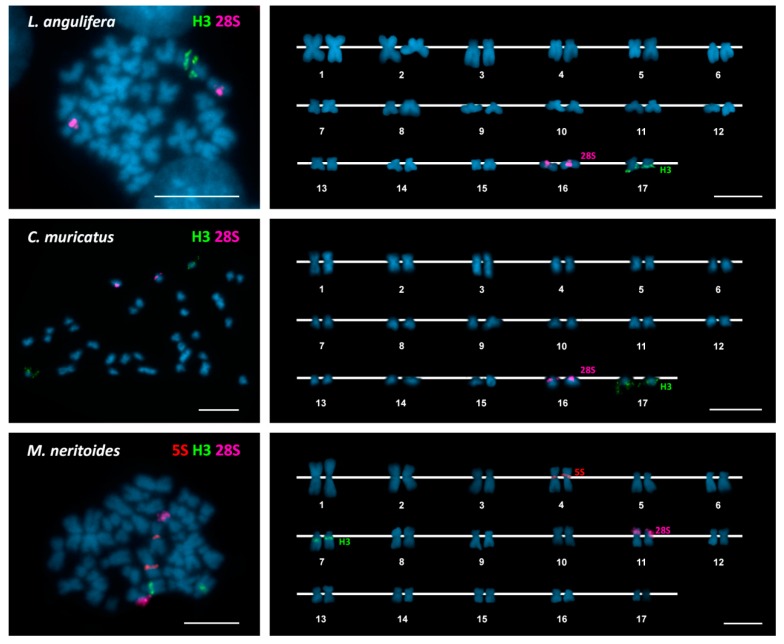
Chromosomal mapping of rDNA and histone gene clusters in mangrove, beaded, and small periwinkles. Fluorescent in situ hybridization mapping of *H3* histone gene (H3, green) and major rDNA (28S, magenta) probes to mitotic chromosomes of the mangrove periwinkle *Littoraria angulifera*, the beaded periwinkle *Cenchritis muricatus*, and the small periwinkle *Melarhaphe neritoides* counterstained with 4′-6-diamidino-2-phenylindole (DAPI). Minor rDNA (5S, red) signals were also detected in the small periwinkle. Scale bars, 5 μm.

As can be seen in Figure 1, Figure 2 and Figure 3, FISH mapping of 28S rDNA probes showed hybridization signals in the nine periwinkle taxa. Five of them displayed a single major (45S) rDNA cluster located at subterminal positions on the short arms of submetacentric chromosome pair 8 in the flat periwinkles *L. obtusata* and *L. fabalis*, subtelocentric pair 16 in the mangrove periwinkle *L. angulifera* and the beaded periwinkle *C. muricatus*, and metacentric pair 11 in the small periwinkle *M. neritoides*. In contrast, two clusters were detected at subterminal positions on chromosome pairs 11 and 15 in the rough periwinkles *L. arcana*, *L. saxatilis*, and *L. compressa*, and on chromosome pairs 8 and 16 in the common periwinkle *L. littorea*.

In regard to *H3* histone genes, a single cluster was detected in all periwinkles: close to the centromere on the long arms of chromosome pair 7 in the rough periwinkles *Littorina arcana*, *L. saxatilis*, and *L. compressa* and the flat periwinkles *L. obtusata* and *L. fabalis*; intercalary to the long arms of chromosome pair 7 in the common periwinkle *L. littorea* and the small periwinkle *M. neritoides*; and at a subterminal location on the long arms of chromosome pair 17 in the mangrove periwinkle *L. angulifera* and the beaded periwinkle *C. muricatus*.

In contrast, 5S rDNA hybridization signals were only detected in the flat periwinkles *L. obtusata* and *L. fabalis* and the small periwinkle *M. neritoides*. In all cases, the single 5S rDNA cluster was close to the centromere on the long arms of chromosome pair 4.

Double-color FISH using 28S rDNA and *H3* histone gene probes labeled differently, followed by rehybridization with a 5S rDNA probe in *L. obtusata*, *L. fabalis*, and *M. neritoides*, demonstrated that all clusters were located on different chromosome pairs in each of the nine periwinkles (Figure 1, Figure 2 and Figure 3). A summary of the FISH mapping results, including those previously published for the small periwinkle *M. neritoides* [14], is presented in Table 3.

## 4. Discussion

Chromosome studies of gastropod mollusks are scarce [29]. This is mainly due to low mitotic indices, small chromosome sizes, and technical difficulties in obtaining good chromosome morphology and spreading. This is particularly evident in Littorinidae, where chromosome numbers were described for only eleven (Table 1) of the around 200 species currently included in the family. The diploid chromosome numbers of 2n = 34 presented by the nine species studied herein, six of them newly described, are coincident with those proposed for seven of the species previously studied and seem to confirm that this is the common number in periwinkles. In contrast, this number differs with those proposed for *Echinolittorina hawaiensis* (2n = 30), *E. miliaris* (2n = 36), *E. radiata* (2n = 36), and *E. subnodosa* (2n = 16) [5,13]. Although those differences could indicate variations in chromosome numbers among different genera in the family, as the divergent diploid numbers (30 and 36) for three of these species were determined from paraffinned material in a study performed in the early 1960s [5], it would be necessary to confirm them using modern techniques of chromosome preparation. In regard to the highly divergent diploid chromosome number of 2n = 16 proposed for *E. subnodosa* [13], it is important to take into account that gastropod mollusks are usually intermediary hosts for digenean parasites, many of which present chromosome numbers and karyotypes [30] similar to the one reported for *E. subnodosa* [13]. Thus, future studies are needed to confirm the chromosome number in this species.

Regarding chromosome morphologies, the superabundance of metacentric and submetacentric chromosome pairs (15 to 17) in the karyotypes of the nine periwinkles described in this work is partially concordant with previous results. The minor discrepancies in karyotype composition can be attributed to differences in the degree of condensation of the chromosomes and the methodology employed [15].

In contrast with previous reports describing differences in chromosome number for male (X0, 33 chromosomes) and female (XX, 34) *M. neritoides* [9,11] and in chromosome morphology for male (XY) and female (XX) *L. saxatilis* [12], we did not detect any difference in chromosome number nor karyotype composition between males and females in any of the nine periwinkle species studied here. In this sense, targeted resequencing in *L. saxatilis* [31] did not reveal any regions with low heterozygosity in males, as expected for an XY sex determination system [32], in agreement with our results. The absence or presence of sex chromosome determinism in these species may have some evolutionary implications. For example, according to Haldane’s rule, intrinsic genetic incompatibilities should affect the heterogametic sex [33]. However, if there are undifferentiated sex chromosomes in *L. saxatilis*, both males and females should be equally affected by incompatibilities. A small degree of postzygotic isolation was observed in hybrid males after the analysis of sperm quality [34], but other postzygotic effects (embryo abnormalities) were also detected in females with intermediate ecotype characteristics [35]. The observation of postzygotic effects in both male and female hybrids is compatible with the absence of differentiated sex chromosomes we found here.

Pertaining to the FISH mapping results, all major (45S) rDNA clusters detected in periwinkles were subtelomeric, but their number differed between one in flat, mangrove, beaded, and small periwinkles versus two in rough and common periwinkles. This variation in the number of 45S rDNA clusters does not completely agree with their phylogenetic relationships (Supplementary materials Figure S1) [1] as the closely related rough and flat periwinkles displayed a different number of major rDNA clusters, whereas the first presents the same number as the more distantly related common periwinkles. Nevertheless, the location of the two clusters differs between the rough and the common periwinkles. The variation in the number of 45S rDNA clusters is a common observation in many taxonomic groups and in some cases has been related to their subtelomeric location. The presence of repeat sequences and breakpoints at subtelomeric regions [36,37], together with clustering of telomeres in meiosis, could favor sequence exchanges between non-homologous chromosomes [38] and consequently explain the variation in number.

Concerning minor (5S) rDNA clusters, hybridization signals were detected in only three of the nine periwinkles studied: the flat periwinkles *L. fabalis* and *L. obtusata* and the small periwinkle *M. neritoides*. In all cases, the single cluster was located close to the centromere in chromosome pair 4. Even though the absence of signals in the remaining six species could be attributable to technical difficulties or a non-clustered nature of the 5S rDNA repeats, the presence of clustered repeats in a number that is below the resolution power of FISH is a more probable explanation.

In contrast to the proposed adjacent location of 45S rDNA and 5S rDNA in the small periwinkle *M. neritoides* [14], the single species of Littorinidae in which FISH was previously applied, our results demonstrated that in both the flat periwinkles *L. fabalis* and *L. obtusata* and the small periwinkle *M. neritoides* the signals for 45S rDNA and those for 5S rDNA appeared in different chromosome pairs.

As commonly reported for other invertebrate groups, we detected a single *H3* histone gene cluster in all nine periwinkles. In contrast, the chromosomal location of the cluster varied from species to species: subcentromeric signals appeared in rough and flat periwinkles, intercalary ones in common and small periwinkles, and subterminal ones in mangrove and beaded periwinkles. The presence of these clusters at subterminal positions is quite unusual in invertebrate species and has only been described in some grasshoppers [39] and bivalves [25,28,40,41,42,43].

Overall, the different probes show the same chromosomal location within flat periwinkles (*L. obtusata* and *L. fabalis*, including three morphotypes/ecotypes in the latter species) and also within the complex *L. arcana*, *L. compressa*, and *L. saxatilis*, in agreement with the monophily of these two groups. However, the differences in the distribution patterns of rDNA and histone gene clusters found in this work indicate that conservation in chromosome number in Littorinidae is not paired by conservation in chromosome structure. This is not a strange phenomenon as demonstrated by the abundance of chromosomal rearrangements (i.e., inversions, translocations) found in sequenced eukaryotic genomes [44,45], including *L. saxatilis* [46].

Although FISH mapping tandemly repeated gene families is a useful tool for chromosome identification in invertebrates, this technique has only been previously applied to map rDNA clusters in a single species of Littorinidae, the small periwinkle *M. neritoides* [14]. In this work, we have demonstrated the feasibility of applying those techniques to locate rDNA and histone gene clusters in nine species of periwinkles, thus opening the possibility of mapping other probes in these organisms and contributing to a better understanding of their chromosome evolution. Even more, the viability of applying FISH to periwinkles also opens the possibility of employing the whole genome of one of the members of a species complex as a probe to hybridize chromosomes of other members of the complex (genomic in situ hybridization, GISH) to obtain insights into their differences and/or to detect interspecific hybridization and introgression.

FISH mapping information has provided valuable information to complement sequencing information for genome assembly in different species. The extension of this technique beyond rDNA and histone gene clusters to increase the contiguity of genome assemblies (chromosome level) and to detect chromosomal rearrangements is likely to shed light on the evolutionary relevance of structural variation and adaptation or speciation in gastropods.

## Figures and Tables

**Table 1:** Haploid (n) and diploid (2n) chromosome numbers and karyotypes in Littorinidae.

Species	n	2n	Karyotype	Reference
*Echinolittorina hawaiensis* (Rosewater & Kadolsky, 1981)	15	30		[5]
*Echinolittorina miliaris* (Quoy & Gaimard, 1833)	18			[5]
*Echinolittorina punctata* (Gmelin, 1791)	16			[8]
	17	34		[11]
*Echinolittorina radiata* (Souleyet, 1852)	18			[5]
*Echinolittorina subnodosa* (Molina, 1782)	8	16	8 m	[13]
*Littoraria strigata* (Philippi, 1846)	17			[5]
*Littorina brevicula* (Philippi, 1844)	17			[5]
*Littorina keena* Rosewater, 1978		34	10 m/sm, 7 st/t	[15]
*Littorina obtusata* (Linnaeus, 1758)		34		[7]
*Littorina saxatilis* (Olivi, 1792)		34	8 m/sm, 9 st/t	[7]
		34	10 m/sm, 7 st/t	[10]
		34	6 m, 9 sm, 2 st	[11]
		34 XX ♀/34 XY ♂	10 m/sm, 7 st	[12]
*Melarhaphe neritoides* (Linnaeus, 1758)	17	34	8 m, 2 sm, 7 t	[6]
	17	34 XX ♀/33 X0 ♂	10 m, 3 sm, 3 st, 1 t	[9]
		34 XX ♀/33 X0 ♂	10 m, 3 sm, 3 st, 1 t	[11]

Notes: m: metacentric; sm: submetacentric; st: subtelocentric; t: telocentric.

**Table 2:** Collection localities of the species of Littorinidae.

Species	Locality	Coordinates
*Littorina arcana*	Holy Island, UK	53.299781, −4.679846
*Littorina saxatilis*	Cabo Estai, Ría de Vigo, Spain	42.182181, −8.813204
	Cabo Silleiro, Spain	42.104987, −8.898929
*Littorina compressa*	Holy Island, UK	53.299781, −4.679846
*Littorina obtusata*	Rande, Ría de Vigo, Spain	42.284311, −8.658661
*Littorina fabalis*	Santo André, Portugal	41.412417, −8.787778
	Praia da Borna, Ría de Vigo, Spain	42.280867, −8.697019
	O Grove, Ría de Arousa, Spain	42.460500, −8.872028
*Littorina littorea*	Marín, Ría de Pontevedra, Spain	42.396351, −8.695599
*Littoraria angulifera*	Miami, FL, USA	25.810107, −80.164063
*Cenchritis muricatus*	Miami, FL, USA	25.889330, −80.150129
*Melarhaphe neritoides*	Cabo Udra, Ría de Aldán, Spain	42.333599, −8.827797

**Table 3:** Chromosomal location of rDNA and histone gene clusters in Littorinidae.

Species	2n	45S rDNA	5S rDNA	*H3* Histone Genes	Reference
*Littorina arcana*	34	11p ter (st)		7q cen (sm)	This work
		15p ter (sm)			
*Littorina saxatilis*	34	11p ter (sm)		7q cen (sm)	This work
		15p ter (sm)			
*Littorina compressa*	34	11p ter (sm)		7q cen (sm)	This work
		15p ter (sm)			
*Littorina obtusata*	34	8p ter (sm)	4q cen (m)	7q cen (m)	This work
*Littorina fabalis*	34	8p ter (sm)	4q cen (sm)	7q cen (sm)	This work
*Littorina littorea*	34	8p ter (m)		7q ic (sm)	This work
		16p ter (sm)			
*Littoraria angulifera*	34	16p ter (st)		17q ter (m)	This work
*Cenchritis muricatus*	34	16p ter (st)		17q ter (st)	This work
*Melarhaphe neritoides*	34	11p ter (m)	4p cen (m)	7q ic (st)	This work
	34/33	p ter (m) *			[14]

Notes: p: short arm; q: long arm; cen: subcentromeric; ic: intercalary; ter: subterminal; (m): metacentric; (sm): submetacentric; (st): subtelocentric; (t): telocentric; *: unidentified chromosome pair.

## Supplementary Materials

The following are available online at http://www.mdpi.com/2073-4425/9/11/517/s1, Figure S1: Maximum likelihood tree based on partial mitochondrial *COI* gene sequences of nine periwinkles using the TN93 + G + I nucleotide substitution model.
