# Supplementary material for: Karyotype Characterization of Nine Periwinkle Species (Gastropoda, Littorinidae)
Source: Genes (Basel). 2018 Oct 23;9(11):517. doi: 10.3390/genes9110517 (PMC6266005; doi:10.3390/genes9110517)
Supplement: Supplementary file 1 [file genes-09-00517-s001.pdf]

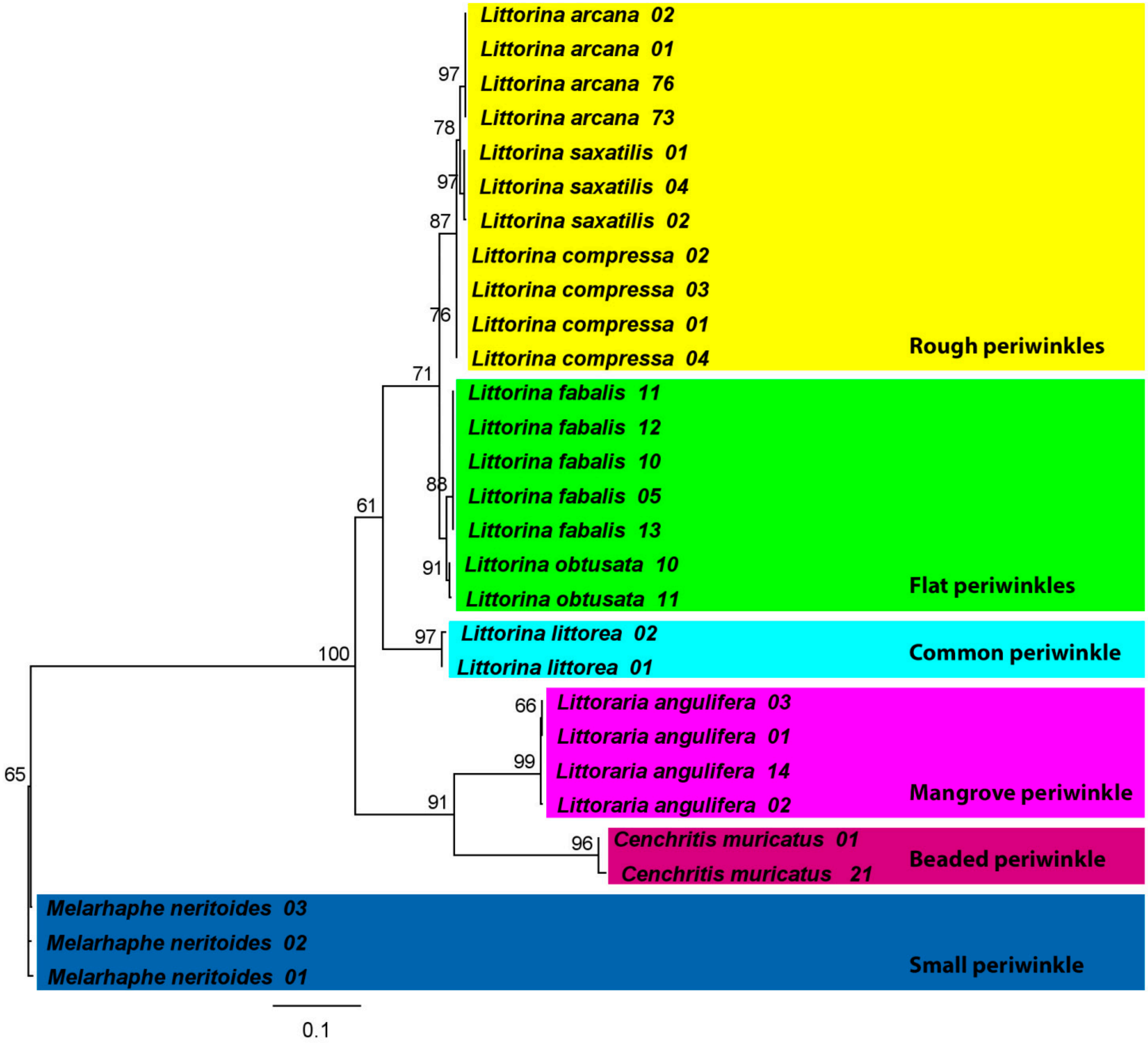

Supplementary Figure S1. Maximum likelihood tree based on partial mitochondrial COI gene sequences of nine periwinkles using the TN93+G+I nucleotide substitution model. Node numbers indicate the maximum-likelihood bootstrap support values >60% (500 replicates). PCR COI products obtained using universal primers [47] were purified (FavorPrepTM GEL/PCR Purification Kit, Favorgen) and sequenced (CACTI, University of Vigo) in both directions in an ABI PRISM 3730 Genetic Analyzer (Applied Biosystems) using a BigDye Terminator v3.1 Cycle Sequencing Kit (Applied Biosystems). Sequences were edited and aligned using Geneious V 11.1.5 (http://www.geneious.com) [48] and subsequent similarity searches against the NCBI nucleotide collection (https://www.ncbi.nlm.nih.gov/nucleotide/) database were performed using the Basic Local Alignment Search Tool algorithm (BLAST), available at the National Center for Biotechnology Information (NCBI, http://www.ncbi.nlm.nih.gov/blast) and the MegaBLAST algorithm set to default parameters. All sequences were deposited in the NCBI GenBank database under the accession numbers MH809396 to MH809424. Analyses were performed with PhyML 3.0 [49] on Geneious V 11.1.5 [48]. Although the tree is based on partial mitochondrial COI gene sequences of nine species, its topology is the almost the same as those previously published [1].
